# The tropmyosin specific IgE and its roles of cross-reactivity between shrimp and dust mites

**DOI:** 10.1186/2045-7022-1-S1-P70

**Published:** 2011-08-12

**Authors:** En-Chin Liao, Mey-Fann Lee, Jaw-Ji Tsaii

**Affiliations:** 1 Taichung Veternans General Hospital, Taichung, Taiwan

## 

Tropomyosin has been reported to be responsible for the cross-reactivity between shrimp and dust mites and the measurement of tropomyosin specific IgE was found superior to shrimp extract for the predicting of shrimp allergic reaction. A total of 55 dust mite allergic patients were recruited to analyse the cross-reactivity between shrimp and mites . Tropomyosin specific IgE for shrimps (rPen a1, nPen i1, nPen m1 ), Anisakis ( rAnis 3 ), house dust mites ( rDer p10 ) and german cockroach ( nBla g7 ) were measured using Phadia diagnotic test. Two recombinant allergens ( rTyr p10 for Tyrophagus putresstienciae and rPer a 7 for American cockroach ) were used to investigate the cross-reactivity . Basophil histamine release (BHR) assay was used to evaluate its biological activities. The results showed that there were 13 patients ( 11 cases of atopic dermatitis AD, 4 cases of bronchial asthma BA and 2 cases of BA and AD) sensitive to tropomyosin. The allergen specific tropomyosin were further analysed, there were 69% (9/13) sensitive to rAnis 3, 62%(8/13) sensitive to rDer p10, 54%(7/13) sensitive to nBla g7 and nPen m1. Most patients were sensitive to at least two different species of tropomyosin. Immuno-Dot study showed that there were 8/13 sensitive to rPer a7 and 7/13 sensitive to rTyr p10. Despite the percent inhibition of BHR were similar between Der p and shrimp stimulation the relationship between the tropomyosin sensitive of all species allergens were not correlated.

In conclusion, tropomyosin specific IgE can be detected in 23.6% (13/55) of Der p sensitive patients and the percentage of shrimp specific tropomyosin allergy was higher in AD than BA. The poor relationship of the specific IgE between the shrimp specific tropomyosin with other species of allergen indicated that the allergenic component of torpomyosin from all allergen should be generated and used for the definite diagnosis of food allergy.

